# Long-Term Effects of Home-Based Family Therapy for Non-responding Adolescents With Psychiatric Disorders. A 3-Year Follow-Up

**DOI:** 10.3389/fpsyg.2020.475525

**Published:** 2020-10-23

**Authors:** Egon Bachler, Benjamin Aas, Herbert Bachler, Kathrin Viol, Helmut Johannes Schöller, Marius Nickel, Günter Schiepek

**Affiliations:** ^1^Institute of Synergetics and Psychotherapy Research, University Hospital of Psychiatry, Psychotherapy and Psychosomatics, Paracelsus Medical University, Salzburg, Austria; ^2^Psychosomatics and Psychotherapy, LMU Ludwig Maximilians University Munich Hospital for Child and Adolescent Psychiatry, Munich, Germany; ^3^Medical University Inssbruck Institute for General Medicine, Innsbruck Medical University, Innsbruck, Austria; ^4^Clinic for Psychiatry and Psychotherapeutic Medicine, Medical Univerity Graz, Graz, Austria; ^5^Department of Psychology, Ludwig Maximilian University of Munich, Munich, Germany

**Keywords:** follow-up, effect size, reliable change index, outcome, adolescents’, family therapies, multi-problem families, Child Behavior Checklist

## Abstract

**Objective:**

Home-based treatment of families with low socio-economic status and multiple psychosocial problems (multi-problem families, MPFs) is gaining importance in clinical social epidemiology and health services research. The sustainability of the treatment is of special importance in order to breach transgenerational effects.

**Methods:**

We examined outcome, effect size, and clinical significance of home-based treatment for 84 multi-problem families in a naturalistic setting. 48 of the families were available for a follow-up after 3 years. The baseline characteristics of these family systems included low collaboration, an increased family adversity index, minors with high rates of child psychiatric disorders, a high prevalence of comorbidity, low relational family functioning, and adolescents who refused any form of treatment or had unilaterally terminated different forms of treatment before. The home-based family therapy consisted of one or two face-to-face counseling sessions per week over an average of 28.8 months (*SD* = 19.2). The symptoms and competence of the adolescents, the caregivers, and the family structure were assessed with 13 variables.

**Results:**

All variables showed significant improvement rates (pre- vs. post- treatment) with medium to high effect sizes (mean of Cohen’s *d* = 1.04, range = 0.34 – 2.18). All variables showed a sustained or even further improvement at follow-up.

**Conclusion:**

This study provides evidence of statistically (p), practically (d), and clinically (RCI) significant changes in symptom and competence-related variables among adolescents and caregivers in MPFs with sustainable long-term effects in the 3-year follow-up period.

## Introduction

### Multi-Problem Families

Multi-problem families (MPFs) are families who experience a multitude of complex problems in various areas of life. Their difficulties usually arise on the level of the family system (psycho-social factors) as well as in their environment (low socio-economic status) (Tausendfreund et al., 2016; Bachler et al., 2018). They range from parenting issues, psychiatric problems, troubled relationships, to financial debt, health-, and housing-related issues, as well as repeated contact with social authorities or the criminal justice system (Tausendfreund et al., 2016). It has been stressed that the difficulties these families experience in their attempt to handle everyday life originate in the interaction between socio-economic and psycho-social problems (Tausendfreund et al., 2016). With regard to the mental health of the minors in these families, several issues are commonly reported in these families (Egle et al., 2004; McLaughlin et al., 2012; Coore Desai et al., 2017): Maladaptive functioning of the parents, disrupted parenting and attachment behavior, associated deprivation conditions, inadequate educational methods, and deficient cognitive, social or emotional developmental support; sometimes even neglect, abuse, and maltreatment. According to Kessler et al. (2010), parental mental illness, child abuse, neglect, and maladaptive functioning of the parents are the strongest predictors of mental disorders, accounting for the occurrence of 29.8% of all disorders in 21 countries. In MPFs, these childhood adversities are highly prevalent, interrelated, and associated with impaired family functioning (Bachler et al., 2018).

### Treatment of MPFs Families

Therapists and social workers aiming to help MPFs families are confronted with a complex system with several levels of dysfunction, spanning psychological, neurobiological, social, and economic issues. This poses a serious challenge for the development of successful therapeutic principles in the treatment of MPFs (Bachler et al., 2017). For a long time, it was assumed that parents and adolescents in MPFs were unable or unwilling to collaborate in a goal-directed manner; they were referred to as “unwilling, involuntary or mandated clients” (Bachler et al., 2016). In the categorization of Friedlander et al. (2011), who identified four groups of individuals or families in psychotherapy (“customers,” “plaintiffs,” “visitors,” and “hostages”), MPFs are found in the “plaintiffs,” “visitors,” and “hostages” categories. These groups are characterized by low problem perception and tend to experience problems externally, which greatly affects the degree of goal-directed collaboration. Collaboration and treatment alliance, however, are the strongest predictor of outcome in psychotherapy (Polaschek and Ross, 2010). The therapeutic alliance and treatment expectancy are impacted negatively (Hamilton et al., 2011) by the main characteristics of caregivers and adolescents in MPFs, including general deficits in social skills, poor object relation, a history of poor familial relationships, strong defensive attitudes, a hopeless stance, low psychological mindedness, high level of resistance, negativism, and hostility (Castonguay and Beutler, 2006). Moreover, pre-treatment characteristics like low socio-economic status, Axis II disorders, and previous non-response are usually predictive of completing the psychotherapeutic treatment without significant clinical improvements (Reuter et al., 2016).

Multi-problem families received relatively little attention in psychotherapy research or in the research on child and adolescent health prior to the 1990s. This was highlighted in a bibliographical survey by Ensminger and Fothergill (2003), who report that only 1% of 11,505 articles between 1990 and 2000 in the Child Development Journal addressed families with low socio-economic status. In the last 15 years, different empirical studies about various forms of home-based treatments in the fields of youth welfare and child and adolescent psychiatry show medium-to-high effect sizes for the treatment of MPFs families, despite multiple chronified psychosocial problems (Curtis et al., 2004; Liddle et al., 2009; Bachler et al., 2016; Tausendfreund et al., 2016). Assuming an average effect size of (*d*) = 0.51 for family therapy (Sydow et al., 2007), an effect size of.55, as found for the Multisystemic Therapy (Curtis et al., 2004), shows the potential of successfully reaching the families. Still, the range of effect sizes (0.27–0.77) reported by Liddle et al. (2009) for Multidimensional Family Therapy suggest considerable differences in treatment response. Preferably, the therapeutic work is done in the home of the families (“in-home” or “home-based” treatment). This setting helps reducing the high dropout rates of 40% within the first 2 weeks for community mental health services (Principe et al., 2006).

### Long-Term Effects of Treatment of Multi-Problem Families

The psychological disorders/problems in MPFs are of great sociopolitical importance as they imply considerable costs for society. These include “undefined burden” like the loss of productivity or inability to work, and “hidden burden,” e.g., delaying treatment and healing through revolving door effects, and worse social networks (WHO, 2001). However, Bachler et al. (2018) showed that childhood adversities do not predestine children to an irreversible fate: The outcome of therapy is more powerful than the influence of adverse factors on the development of child psychiatric symptoms. Enduring effects of the treatment are of high relevance for both the individual and the society.

While the feasibility of effective treatment of MPFs has been elaborated in the previous paragraphs, the long-term effect of these treatments has hardly been in the focus of research. A follow-up on child psychiatric treatments has been examined for only a few clinical groups of adolescents, such as those with bipolar disorders (Inder et al., 2017), former adolescent self-injurers (Groschwitz et al., 2015), or in cases of anorexia nervosa (Amianto et al., 2017). Amianto et al. (2017) demonstrated that 8 years after treatment, the rate of complete remission was 42, 67.8% improved, and 18.6% worsened. The long-term effects of home-based treatment have been examined with regard to drug abuse and delinquency. van der Pol et al. (2018) found that 3 years after treatment, delinquency and cannabis abuse had significantly declined, although no difference was found between multidimensional family therapy and cognitive behavioral therapy. In another study, dealing with juvenile sexual offenders, the effects of multisystemic therapy were more sustained than for group cognitive behavioral therapy (Letourneau et al., 2013). Johnides et al. (2017) examined the long-term effect of multisystemic therapy on caregivers of serious juvenile offenders in a 20-year follow-up. Caregivers in multisystemic therapy showed 94% fewer felonies and 70% fewer misdemeanors than caregivers in the individual therapy setting.

### Aims and Hypotheses

This study is part of a research project on empirically supported principles in the treatment of MPFs families (“What works for whom and why?”). Here, we assess the effects of the home-based Outpatient Family Therapy (OFT) on a variety of psycho-social factors, including a follow-up analysis to examine the stability of the treatment effects after 3 years. The research questions were as follows: (1) Does OFT improve the functioning, mental health, and social abilities of the clients? (2) Do these effects sustain 3 years after the treatment? The analysis plan differentiates between self-rating measures (behavioral and psychiatric assessment of the minor) and external ratings (other variables).

(1) Self-rated variables
  (a) OFT will decrease the problematic behavior of the minor.
  (b) OFT will decrease the psychiatric symptoms of the minor.
(2) Externally rated variables
  (a) OFT will increase the global functioning of the minor, the caregiver, and of the family.
  (b) OFT will increase the social and working skills of the minor and the self-sustainability of the family.
  (c) OFT will decrease general risks (adversities) of the family.
  (d) OFT will increase the achievement of individual therapeutic goals.

## Materials and Methods

### Study Design

This is a longitudinal study with a naturalistic design. The study was conducted by a research group from two institutes, the Institute of Psychoanalysis and Family Therapy (IPT) in Salzburg, Austria, which operates in two Austrian federal states and in Upper Bavaria, Germany, and the Institute of Synergetics and Psychotherapy Research of the Paracelsus Medical University, Salzburg, Austria. The study was part of a larger research project about the treatment of MPFs funded by the EU.

The Youth Welfare Office regularly puts families forward to the Institute of Psychoanalysis and Family Therapy for treatment if other treatments have failed or have been refused, if a child or adolescent is threatened with placement in a therapeutic institution, or if the court has ordered therapeutic intervention. Overall, the families show a wide range of psychosocial problems and imminence of various forms of child endangerment. Therapy was provided randomly by 34 therapists (21 female, 13 male) out of a total of 170 psychotherapists of the Institute of Psychoanalysis and Family Therapy, servicing 650 families at any one time. The 34 therapists who treated the families of this study were responsible for an average of 2.4 families (*SD* = 1.3). They knew that their case was part of an empirical study but did not know the hypotheses of the study.

The therapists assessed the collaboration and the families’ treatment expectancy as part of the routine assessment (for details see Measures). Additional data were collected by external clinical psychologists in the families’ homes before and after treatment, and 3 years after the treatment (follow-up). The external clinical psychologists had received training in data collection and were not employs of either institute.

Confidentiality was guaranteed and written informed consent was provided by all participants (or their legal representatives) according to the Declaration of Helsinki.

### Measures

In the following, we provide basic information on the questionnaires and assessments used in the study. Details can be found in the supplement. Six measures were applied before and after treatment: collaboration, treatment expectation, three individual therapeutic goals (ITG), and the mental health of the minor. Seven measures were also assessed three years after treatment (follow up): behavior of the minor, family adversities, relational functioning within the family, global functioning of the minor, global functioning of the caregiver, social self-sustainability of the family, and social/working skills of the minor.

#### Self-Rating Measures

##### CBCL

The symptoms and behavior of the minors were assessed by the Child Behavior Checklist (CBCL) and the Mannheim Parental Interview (MPI). The CBCL (Achenbach, 1991) is a widely used international scale and provides information on three domains of competence (activity, social competence, school). In addition to a total score, it comprises eight subscales (somatic, social problems, social withdrawal, anxiety/depression, alertness, schizoid/obsession, dissocial behavior, aggressive behavior), and allows for the assessment on externalizing and internalizing behavior.

##### MPI

The MPI (Esser et al., 1989) is a structured and standardized clinical interview, which indicates psychological disorders and their severity. The 37 questions covering child and adolescent psychiatric symptoms combine a cumulative child-psychiatric symptom score and different ICD diagnoses.

#### Externally Rated Measures

##### CP and VH

The assessment of the collaboration (Collaboration Scale, CP, Bachler, 2013) and treatment expecation (Treatment Outcome Expectation, VH, Bachler, 2013) are integral parts of routine assessments. Collaboration is assessed by a narrative interview which the therapist conducts with the family. The therapist then estimate the degree of collaboration with the family reaching from 1 (excellent) to 5 (impossible). The Treatment Outcome Expectation is rated by the therapist together with the caregiver and the adolescent ranging from 1 (high) to 5 (low) expectations.

##### ITGs

The ITG rating follows the ITG module of the Psychotherapy Basic Documentation (PSYBADO, Heuft and Senf, 1998). It provides an individual definition of three therapeutic goals that are important both to the family and the Child Welfare Office and rated independently by the family, the external psychologist, and the Welfare Office. The final score is the average of the ratings.

##### FAI

The Family Adversity Index (FAI) (Rutter and Quinton, 1977) measures familial psychosocial stress. Based on five items (chronic disharmony in the family, low socioeconomic status, cramped living quarters, parental criminality, and mental disorder of the primary caregiver), the resulting total value ranges from zero to a maximum of five. Values ≥2 reflect considerable socio-familial stress. The FAI is rated by the external psychologist based on the anamnestic information they received.

##### SSF/SSAMJ

The Social Self-Sustainability Skills Scale (SSF) records the social self-preservation ability ranging from 1 (very good) to 5 (massively restricted). The SSF describes – independently of social support systems – factors such as social assistance, working capacity, and the family income earned through this work. The SSAMJ records the school and work ability of the minors and defines the extent to which age-appropriate social behavior and performance can be achieved (Bachler, 2013). SSF and SSMAJ are rated by the external psychologist.

##### GAF/CGAF/GARF

The Global Assessment of Functioning scale, based on the DSM-IV, is frequently employed in psychotherapy studies as a measure of disability and psychosocial dysfunction (Saß et al., 2003). The questionnaire comes in an adult version (GAF) and a version for children aged 4 and above and adolescents (CGAF). The Global Assessment of Relational Functioning (GARF) rating scale assesses the psychosocial level of family functioning through a clinical interview. It covers the three dimensions problem solving, organization, and emotional climate (Stasch and Cierpka, 2006). The three questionnaires are rated by the external psychologist.

### Sample

#### Calculation of Sample Size

Prior to the beginning of the study, the number of samples needed was calculated. The lowest effect size in former research (Bachler et al., 2016) was *d* = 0.35. To achieve a power of.8, 66 subjects were needed to get a significant result for a two-sided paired *t*-test (α = 0.05). We decided to collect data from 90 families due to the unknown dropout rate in the follow-up period.

#### Definition of the Sample

The sample was defined consecutively, i.e., all families put forward with a treatment order by the Youth Welfare Office starting from 4/2008 were included in the study, unless they met an exclusion criteria (see below). The probability of being included in the representative random sample was therefore the same for each family as required by the Equal Probability Selection Method.

**FIGURE 1:**
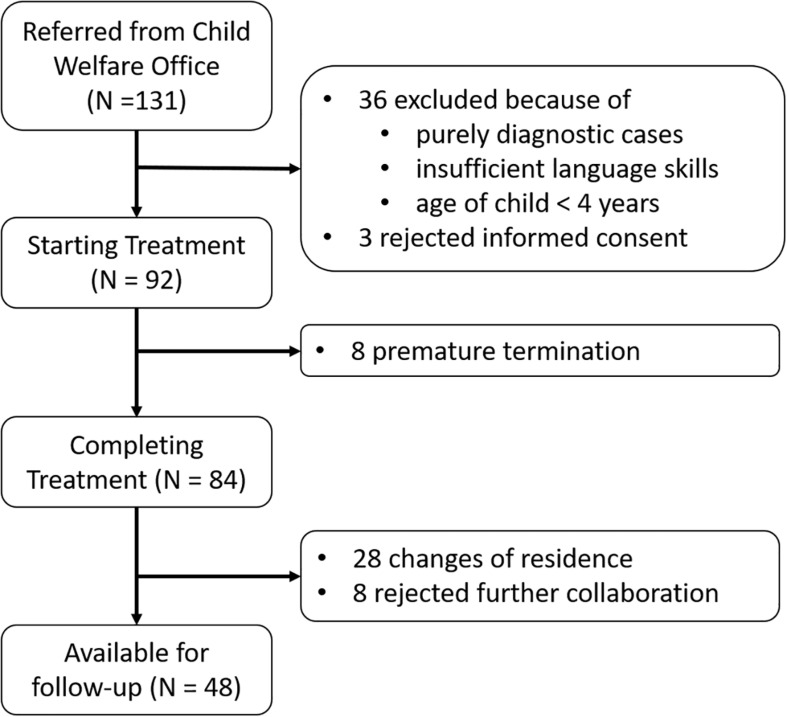
Flow chart showing the composition of the resulting sample. OFT: outpatient family therapy. For the pre vs. post comparison, 84 families were available; for the differences to the follow-up, data of 48 families could be reached.

From the 131 families contacted during the study period (Figure 1), 36 cases were excluded because they were either purely diagnostic clarification cases (clearing), or the caregivers had insufficient language skills, or the age of the child at the start of treatment was below 4 years. Further three families refused to give written informed consent due to data protection reasons related to the Youth Welfare Office. The treatment of 8 families was prematurely terminated within the first 24 weeks of therapy. In consequence, the final sample size for the pre vs. post treatment assessments was 84. Out of these 84 families, 48 also participated in the follow-up (28 families could no longer be contacted due to a change of residence, and 8 declined to collaborate further). Corresponding to the intention-to-treat principle, families that discontinued the treatment after 6 months (long-term) were nevertheless included in the evaluation.

All the adolescents were so-called non-responders, i.e., they had refused treatment or had received different forms of treatment with early dropout (within the first 2 months). None finished treatment voluntarily.

#### Sample Characteristics

The mean age of all clients that were approached for follow-up measurements was 10.65 years (range = 4–17; median = 10.5; *SD* = 3.52), with 29 female and 56 male clients and a mean treatment duration of 28.8 months (*SD* = 19.2). The mean age at follow-up was 14.0 years (range = 7–20). All children were diagnosed with mental illness (Table 1), mostly with behavioral and emotional disorders beginning in childhood and adolescence (F90-98; ICD 10, 2006). They were coded according to the Mannheim Parent Interview (MPI) (Esser et al., 1989) into four spectra (rounded percentages): 34% were diagnosed with dissocial disorder, 26% with emotional disorder, 27% with hyperactivity, 6% with alcohol and drug abuse/dependency, and 6% with other ICD-10 diagnoses (F84, F95, and F98). More than half of the children (*N* = 58) had comorbidities; 31 of the adolescents were assigned to two, and 27 to more diagnoses. The mean of the Child Behavior Check List (CBCL, see Measures and Table 2) total score was 48.5 (*SD* = 23.7). The transformed *T*-value (*T* ≥ 67) of our sample is at the 95–98% percentile, i.e., only 2% of the children in the total population show such high values. The *T*-value is nearly two standard deviations above the mean of the standard sample (*T*-value 50, 1 SD = 10), with a *T*-value above 1 standard deviation representing the cut-off value between healthy and sick (Achenbach, 1991).

**TABLE 1:** Demographics, diagnostics, and pre-symptom scores of the sample.

**Characteristics**	***M* (*SD*) or%**
*Family*	
Treatment duration (month)	28.8 (19.2)
Very low SES	37%
Chronic Disharmony	72.4%
*Child/adolescent*	
Age	10.65 (3.52)
Sex	34.0% female
ICD diagnosis	100%
Alcohol and drug addiction	6%
Emotional disorder	26%
Dissocial disorder	34%
ADHD	27%
other diagnoses	6%
Comorbidity	69%
*Primary caregiver*	
Drug addiction	19.7%
Uneducated	29.4%
Mental disorder	63%
Age	43.6 (6.1)
Sex	93.6% female
Single parents	53.2%

*M, mean; SES, socio-economic status; SD, standard deviation.*

**TABLE 2:** Pre-treatment scores, post-treatment scores, and follow-up scores of all measures with the effect sizes of the changes.

	**Pre-treatment**			**Post-treatment**				**Follow-up**		**Effect sizes of change (Cohen’s d)**		
										**pre vs. post**	**pre vs. follow-up**	**post vs. follow-up**
Variable/Measure	**M**	**SD**	**95% CI**	**M**	**SD**	**95% CI**	**M**	**SD**	**95% CI**	**d**	**d**	**d**
Collaboration (CP)^t^	2.99	0.95	[2.8;3.2]	2.25	1.04	[2.0;2.5]				0.74**		
Treatment expectation (VH)^t^	3.44	1.10	[3.2;3.7]	2.83	1.11	[2.6;3.1]				0.55**		
Individual therapeutic goal 1 (ITG-1)	−1.58	0.61	[−1.7;−1.5]	0.33	1.08	[0.1;0.6]				−2.18**		
Individual therapeutic goal 2 (ITG-2)	−1.55	0.59	[−1.7;−1.4]	0.19	0.98	[−0.02;0.4]				−2.14**		
Individual therapeutic goal 3 (ITG-3)	−1.56	0.61	[−1.7;−1.4]	0.18	0.95	[−0.03;0.9]				−2.17**		
Mental Health of the child (MPI)	13.70	8.24	[12;16]	7.93	8.13	[6.2;9.7]				0.67**		
Family adversity index (FAI)	2.24	1.13	[2.0;2.5]	1.85	1.08	[1.6;2.1]	0.92	0.83	[0.7;1.2]	0.35**	1.28**	0.91**
Global functioning of the caregiver (GAF)	59.88	1.48	[5.6;6.3]	67.98	1.60	[6.5;7.2]	78.09	1.65	[7.3;8.3]	−0.52**	−1.13**	−0.59**
Global functioning of the minor (CGAF)	53.48	1.49	[4.9;5.8]	68.48	1.93	[6.3;7.4]	78.51	1.53	[7.4;8.3]	−0.86**	−1.40**	(−0.34)
Relational functioning of the family (GARF)	24.13	0.83	[2.2;2.7]	34.78	0.94	[3.2;3.8]	40.85	0.99	[3.8;4.4]	−1.19**	−1.81**	(−0.37)
Child behavior checklist (CBCL)	48.52	23.71	[43;54]	29.58	20.07	[25;34]	24.63	19.4	[19; 30]	0.86**	1.22**	(0.20)
Social self-sustainability (SSF)^t^	3.14	0.98	[2.9;3.4]	2.79	1.12	[2.5;3.0]	2.48	1.20	[2.1;2.8]	0.34*	0.44*	(0.08)
Social and Working Skills of the minor (SSAMJ)^t^	3.85	1.00	[3.6;4.1]	2.76	1.19	[2.5;3.0]	2.48	1.13	[2.2;2.8]	0.98**	1.22**	(0.12)

*: p < 0.05 **: p < 0.001 of the paired t-test, corrected for multiple comparisons. ^t^ inverted scale, e.g., high scores = low expectations.

The primary caregivers were between 32 and 55 years of age (*M* = 43.6; *SD* = 6.1). 93.6% of the primary caregivers were female. The rate of single parents (divorced, widowed, separated, living alone) was 53.2%. The proportion of primary caregivers with mental illnesses was 63% (29–35% were personality disorders with a moderate to disintegrated structure level).

The families showed a high level of vulnerability to socio-familial burdens: Social problems such as poverty and unemployment as measured by the FAI (FAI, see Measures and Table 2) at the start of care was *M* = 2.24. According to Rutter and Quinton (1977), the cut-off value for a “significant socio-familial burden” is ≥2. Likewise, the social functioning of the family was low, as indicated by the GARF (Stasch and Cierpka, 2006).

### Outpatient Family Treatment

The treatment method applied in this study is therapeutic Outpatient Family Treatment (OFT), which was developed as a disorder-oriented, therapeutic outreach intervention for families with multiple problems. It combines structural family therapy interventions (Minuchin and Fishman, 1983), psychoanalytic elements of mentalization-based psychotherapy (Fonagy et al., 2006), and structural psychotherapy (Rudolf, 2006). The aim of OFT is to improve the intra-psychological and interpersonal ego-structural skills such as perception of self and others, defense and affect regulation, attachment, and communication (cf. Opd Arbeitskreis 2, 2006, Axis IV) in the primary caregiver and in the minors. Special regard is payed to the general parenting skills of the primary caregivers. The program incorporates the principles for the treatment of personality disorders and structural psychotherapy (i.e., aiming at the improvement of ego-structural competencies) that were identified by the APA Division 12 task force: a strong working alliance, therapist ability to repair alliance ruptures, collaboration on goals, and a high level of therapist activity (Critchfield and Benjamin, 2006). The therapists in this study had different therapeutic backgrounds (36% in psychodynamic therapy, 29% in family therapy, 19% in cognitive-behavioral therapy, 16% in other therapeutic approaches) and had received specific training in the technical characteristics of the OFT approach based on a curriculum. A manual is kept describing the OFT performed in the study. There are obligatory interventions: (A) fostering goal consensus and collaboration by fostering treatment expectancy and giving support to solve problems in order to reduce the impact of adversity factors, (B1) focused work on organization and bonding, communication, and emotional climate in the families, (B2) focused work on individual goals defined with adolescents and parents, (C) the process of completion by continuously decreasing the intensity and frequency of the treatment to foster the autonomy of the adolescents and the functioning of the family system. In addition, the following are important: repair of alliance, management of countertransference, and an interpersonal focus on the improvement of social skills.

The level of therapeutic directiveness and support is initially high and multimodal; treatment length and frequency are adaptive. The average number of therapy hours in the institute and for the sample amounted to 2.5–3 h per week, mostly divided into two sessions. Supervision was given by experienced clinicians who did not belong to the team of therapists once a week.

### Analysis

The collected data were analyzed with SPSS 21.0 and Matlab (R2018b). First, two repeated measures multivariate analysis of variance (rMANOVA) using Wilks-Lambda were performed to assess the combined change of the measures over time. One rMANOVA included the pre/post difference with all measures as dependent variables, and time as the independent factor with two levels (pre/post). The other rMANOVA included only the seven measures where a follow-up score was available. As post hoc tests, paired t-tests were used to test significant differences between pre- and post-treatment scores, between pre-treatment and follow-up scores, and between post-treatment and follow-up scores of each of the measures. The significance level was set to.05 for all tests and the false-discovery rate (FDR) algorithm by Benjamini and Hochberg (1995) was applied using a Matlab toolbox (Fachada and Rosa, 2018).

Cohen’s effect size d was calculated for the mean difference between pre- and post-treatment scores and then divided by the pooled standard deviation σ_pooled_ according to *d*=(*M*_post_-*M*_pre_)/σ_pooled_. Following Morris and DeShon (2002), the pooled standard deviation was calculated by relating the standard deviations of the pre and post treatment values (σ_pre_ and σ_post_) to their correlation r_pre/post_:

$$\sigma_{\text{pooled}}=\frac{\sigma_{\text{pre}}\,\sqrt{2\,\left(1-r_{\text{pre}/\text{post}}\right)}+\sigma_{\text{post}}\,\sqrt{2\,\left(1-r_{\text{pre}/\text{post}}\right)}}{2}.$$

Reliable changes were analyzed using the Reliable Change Index method (RCI; Wise, 2004) according to $\text{RCI}=\frac{M_{\text{pre}}-M_{\text{post}}}{\sigma_{\text{pre}}\,\sqrt{2\,\left(1-\alpha \right)}}$, where α is the reliability of the measure (Cronbach’s α). Applying a 5% criterion, an RCI ≥ 1.96 and RCI ≤ −1.96 thus identified the cases that have reliably changed (*p* < 0.05).

All variables of the Children Behavior Check List were represented by *T*-norms based on German norms.

## Results

### Preliminary Analysis

First, we were interested if there were any differences between the families who were available for the follow up (*N* = 48) and those that were not (*N* = 37). Both groups did not significantly differ in terms of age, gender, collaboration, behavior of the child (CBCL score), or any of the other pre/post change scores, neither at the beginning of treatment nor in the pre/post difference. Only the length of the two groups’ respective treatment was significantly different: families participating in the follow-up had a significantly shorter treatment duration of 24.9 months compared to 34 months for the non-participating families [*F*(1,83) = 4.92; *p* = 0.029]. In addition, Levene’s test showed no violation of homogeneity (except the difference scores of SSAMJ), suggesting no structural differences between the two groups. Overall, the results suggest that the drop-out group did not systematically differ from the follow-up group.

### Improvements During Treatment and Follow-Up

The repeated measures MANOVA of the pre/post scores was significant [*F*(13,32) = 13.14, Λ = 0.16, *p* = 0.001, η^2^ = 0.84], suggesting a difference in the combined dependent variables. Also, the rMANOVA of the variables with follow-up scores revealed a significant change over time [F(14,10) = 6.28, Λ = 0.10, *p* = 0.003, η^2^ = 0.90]. In consequence, paired *t*-tests were conducted to determine which of the variables showed significant changes, and – in the case of the rMANOVA with follow-up scores – where in time the significant changes took place.

**FIGURE 2:**
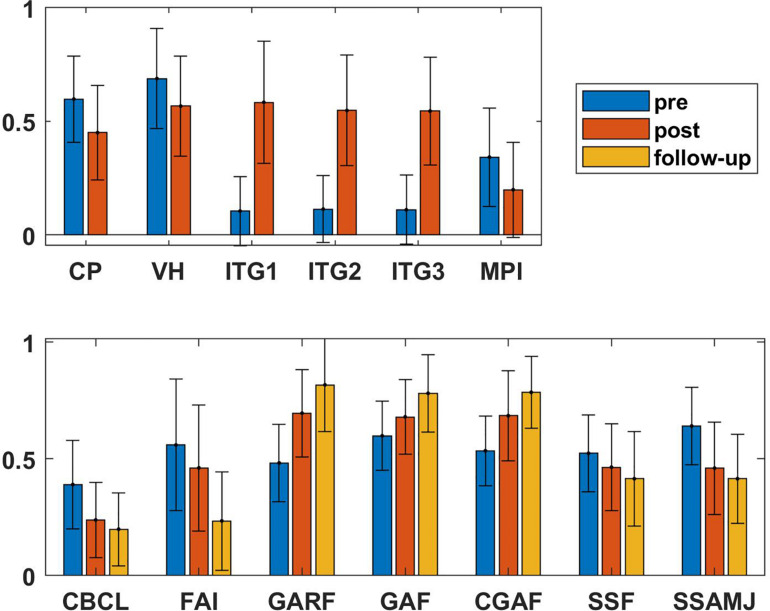
Visualization of the pre, post, and follow-up values of the scores of the multi-problem families. To allow comparability, all values were rescaled by the range of the respective questionnaire, i.e., 1 corresponds to the maximum and 0 to the minimum values. By this, the pre/post/follow-up differences are comparable. Symptom scores like MPI, CBCL and SSAMJ significantly decreased during treatment and continued to decline in the 3-year follow-up period. Accordingly, the scales assessing the functioning of the families increased (GAF, CGAF, and GARF). The measures for general risks of the families (FAI and SSF) decreased, too. The largest change was achieved in the ITG of the families. The scores for treatment expectation and collaboration are reversed, i.e., high score represent low expectations and missing collaboration. CBCL: Children Behavior Check List, CGAF: global functioning of the child, CP: collaboration, ITG: ITG, FAI: family adversity index, GAF: global functioning of the caregiver, GARF: global relational functioning of the family, MPI: Mannheim Parental Interview, SSF: (impaired) Social Self-Sustainability of the family, SSAMJ: (impaired) Social and Working Skills of the Minor, VH: treatment expectation.

As shown in Table 2, all measures showed significant improvements during treatment (pre vs. post) with generally large effect sizes (Cohen’s *d*, *M* = 1.04; range = 0.34 – 2.18). The highest effect sizes were obtained for the three ITG (*d* = 2.1 – 2.2). Without the ITG, the mean effect size was still *d* = 0.70. Importantly, the improvements even continued after treatment: the comparison of scores from before treatment with follow-up scores show a sustained consolidated treatment effect: the mean effect size between pre and follow-up increased to *d* = 0.73 (without ITG). When comparing the outcome measure after treatment with the scores from the follow-up, two of the variables, the global functioning of the caregiver and the FAI, even showed a second significant improvement [GAF: *t*(46) = −3.6; *p* < 0.001; FAI: *t*(46) = 4.8; *p* < 0.001]. Figure 2 summarizes the findings of the key measures, displaying the scaled pre, post and follow-up scores.

Even though the CBCL total score (post-treatment vs follow-up) did not show further significant improvement after treatment, Table 3 shows that the values of the subscales “attention deficits” and “aggressive behavior” significantly decreases further during the 3-year follow-up period. The subscale “somatic problems” *in*creased significantly during the follow-up period. However, the somatic problems did not deteriorate compared to the pre-treatment score.

**TABLE 3:** The table shows the changes of the subscores of the Children Behavior Checklist (CBCL) over time.

	**Pre vs. Post**			**Pre vs. Follow-up**			**Post vs. Follow-up**		
**Variable/Measure**	***M***	***SD***	***d***	***M***	***SD***	***d***	***M***	***SD***	***d***
Social withdrawal	1.32	2.56	0.51**	1.98	2.75	0.75**	0.19	2.02	(0.09)
Somatic problems	0.75	2.22	0.36*	0.14	2.48	(0.07)	−0.58	1.75	−0.40*
Anxious depressive	2.74	3.89	0.65**	4.10	3.92	0.97**	0.65	2.43	(0.19)
Social problems	1.52	2.18	0.59**	2.00	1.98	0.77**	0.52	1.95	(0.23)
Schizoid-obsessive	1.12	1.86	0.69**	1.31	2.05	0.74**	−0.02	1.30	(−0.02)
Attention deficits	2.31	3.62	0.64**	3.33	3.67	0.91**	0.71	2.28	0.22*
Dissocial behavior	1.53	3.06	0.43**	2.17	3.97	0.61**	0.00	3.62	(0.00)
Aggressive behavior	4.88	7.29	0.64**	8.77	7.38	1.22**	2.25	5.39	0.35*
Other problems	3.74	3.93	0.88**	4.17	4.99	0.93**	0.31	2.93	(0.09)
Internalizing	4.55	6.64	0.66**	5.85	7.00	0.88**	0.21	4.28	(0.04)
Externalizing	6.41	9.8	0.61**	10.9	10.5	1.09**	2.25	8.30	(0.25)

N = 84 for the pre vs. post difference, *N* = for the pre and post vs. follow up differences. *p < 0.05, ** *p* < 0.001 d, effect size (Cohen’s d); M, mean; SD, standard deviation.

The development of the minors was assessed by three scales, the CGAF, the MPI, and the CBCL. While the CGAF is based on an external rating, the latter two measures are self-rated (or rated by the parents). In contrast to findings from previous research (Minami et al., 2007), we did not obtain larger effect sizes for the self-report questionnaires (*d* = 0.67 for the MPI and *d* = 0.86 for the CBCL) compared to the externally rated CGAF (*d* = 0.86).

### Clinically Relevant Changes

In order to investigate if the above-mentioned changes were also clinically relevant, the Reliable Change Index (RCI) was computed for the four main measures: CBCL, GAF, CGAF, and GARF. The respective percentages of improved and recovered clients is shown in Table 4. Scores that changed more than 1 SD were categorized as “improvement,” changes larger than −1 SD as “deterioration,” and values between −1 and 1 SD as “unchanged.”

**TABLE 4:** Reliable Change Index (RCI) and the percentages of improved (>1 SD), deteriorated (<−1 SD), and unchanged patients.

		**Pre vs. Post**			**Pre vs. Follow-up**		
**Measure**	***RCI***	**% Improved**	**% Unchanged**	**% Deteriorated**	**% Improved**	**% Unchanged**	**% Deteriorated**
CBCL	25.44	38.8	58.8	2.4	45.8	54.2	0.0
GAF	2.10	8.3	90.5	1.2	38.3	61.7	0.0
CGAF	2.11	21.7	76.1	2.2	54.2	41.7	4.2
GARF	0.69	69.6	26.1	4.3	70.8	29.2	0.0

*CBCL, Child Behavior Check List; CGAF, Global Assessment of Functioning (child); GAF, Global Assessment of Functioning (caregiver); GARF, Global Assessment of Relational Functioning (family).*

The deterioration score for all clinical parameters was low (range: 0 – 4.3%), and all variables showed further improvements in the follow-up period. The global functioning of the caregivers (GAF) showed the highest percentage of an “unchanged status” in the treatment period (91%), while the minors’ rate of unchanged participants was considerably lower (CGAF, 76%). This suggests a higher impact of OFT on adolescents in comparison to their caregivers. The largest improvement rate was achieved for the relational functioning of the families (GARF, 26% unchanged).

Most importantly, there were no deteriorations of the minors in the follow-up period. 45.8% of adolescents with unilateral termination in previous treatments (non-responders) showed clinically significant and sustainable improvement of the total score of the CBCL and CGAF (pre vs. 3-year follow-up) in the setting of the OFT.

## Discussion

This study provides evidence of statistically (*p*), practically (*d*), and clinically (*RCI*) significant changes in symptom and competence-related variables among adolescents and caregivers in MPFs with sustainable long-term effects in the 3-year follow-up period.

### Improvement of Caregivers, the Family System, and Children’s Mental Health (External Rating)

At the beginning of treatment, the relational functioning (GARF) of the families was at a very low level (*M* = 24.1). The effect size of the treatment (pre vs. post) was high (*d* = 1.19) and well above the effect size of *d* = 0.87 reported by Zander et al. (2001). However, the post treatment score was still in the dysfunctional range. Importantly, the relational functioning within the family further improved after treatment, resulting in an unremarkable follow-up score (*M* = 41.0). This shows a sustainable, high improvement of family functioning (competence related improvements in problem solving, family organization, and emotional climate), which is preventive for the further development of siblings and the family as a whole.

These changes might be related to the psychological health of the caregivers (GAF), which improved with a high effect size (*d* = 0.52) from 59.9 to 68.0 and almost reached the cut-off value for health (70.0) after treatment. After three years, the caregiver’s competence scores were within the range of healthy adults. The GAF has an important binding-based mediator function with respect to the relational functioning (GARF) and the mental health of the kids (GAF). Parents with low personality functioning have significant vulnerability-relevant skills-related deficiencies (Bornstein and Bradley, 2003). Mötteli et al. (2018) found in a propensity-score matching analysis of 19 months a change in the GAF for mentally ill adults from 41 to 67 in home treatment. The improvement of mental health of the caregiver was related to a moderate improvement of the self-sustainability of the family (SSF), i.e., the caregiver’s ability to work. The complex interactions between psychological health of the caregivers, family functioning, and the mental health of the minors calls for further empirical research.

In a sample of the same institute that had received the same treatment (start of treatment 2009, end of treatment 2013, *N* = 376, Bachler et al., 2016), the effect sizes (Cohen’s *d*) between pre and post treatment: CP = 0.46 (0.67); VH = 0.53 (0.51); ITG = 1.45 (1.46); FAI = 0.35 (0.45), MPI = 0.47 (0.81); GAF = 0.52 (0.52); CGAF = 0.86 (0.87); GARF = 0.70 (1.04). The effect sizes of the study presented in this manuscript are given in parentheses. The sample of the other group was defined in the same way: randomly, consecutively (i.e., all families put forward with a treatment order by the Youth Welfare Office starting from 4/2008 were included in this study), and with the same exclusion criteria as the present study, but no follow-up data is available for this group.

Taken together, the general improvement of the family is reflected by the decreased risk factors of the families: The FAI, which comprises multiple psycho-social adversities such as chronic disharmony in the family, low socio-economic status, cramped living quarters, parental criminality, and mental disorder of the primary caregiver, decreased significantly. The highest effect sizes were achieved for the ITG (*d* = 2.1 – 2.2), confirming once more the importance of personalized treatment (Schiepek et al., 2016).

Likewise, improvement of the children’s social, school, and work capabilities (SSAMJ) are essential for therapeutic work, success at school and at work, and lead to higher social mobility (Lund et al., 2011).

### Improvement of Children’s Mental Health (Self-Rating)

The problematic behavior of the children (CBCL value) of the mixed-gender group of our study before treatment was clinically conspicuous, while the post value was almost within the normal range. Our study shows moderate to high effect sizes for externalizing disorders (comprising the subscales social problems, dissocial behavior, and aggressive behavior). These changes are of particular importance since Bellani et al. (2012) demonstrated the association between externalizing disorders in minors and Axis I and Axis II disorders in adulthood. Even the three subscales of the CBCL Dysregulation Profile (CBCL-DP), which comprises anxious-depressive, attention deficits, and aggressive behavior, improved with medium effect size. Holtmann et al. (2011) showed that CBCL-DP scores are not only pediatric symptoms, but also provide an early marker for developmental risks, persistent deficits in affect, self-regulation, suicidality and social behavior. Cross-sectional studies show strong empirical evidence of increased rates of children with CBCL-DP suffering as adults 24 years later from disorders of anxiety, mood, personality, and disruptive behavior as well as marked impairment, suicidality, and multiple DSM-IV diagnoses (Reef et al., 2011). The odds ratio is 11.6 that children with a high CBCL-DP are likely to abuse drugs and suffer from severe psychiatric disorders (Althoff et al., 2010; Bellani et al., 2012).

The score for the children’s psychiatric symptoms (MPI) also showed a clear improvement with medium effect-size (*d* = 0.67), The scores of the MPI and the CBCL indicate sustained competence-related improvements of the adolescents. This is of great importance since it allows to interrupt the transgenerational pattern of dysfunctional parenting.

### Practical and Clinical Significance of the Improvements

Reviews and meta-analyses of studies on psychotherapy outcomes show that 5–10% of patients in psychotherapy experience a worsening of symptoms, and 15–25% evidence no clinically significant improvements (Stulz et al., 2007). In a study of 1,776 patients, Jacobi et al. (2011) showed worsening rates of 0.8–4.3% and a high-symptomatic completion value (*M*_*pre*_ + ≤1 *SD*) indicating “no change” in 11.2% of the patients. In our study, we calculated the RCI for the four most important variables (CBCL, GAF, CGAF, and GARF). All showed considerable post-treatment clinical improvements among the participants. The rate of deterioration post-treatment was not higher than in outpatient psychotherapy (Jacobi et al., 2011), but 29.2–61.7% remained unchanged. This indicates differential effects of OFT on different subgroups of MPF as it was shown in previous research (Bachler et al., 2017).

The effect sizes of our study in comparison with different studies on Multi-Systemic Treatment (MST) and Multi-Dimensional Family Therapy (MDFT) demonstrate the following: OFT treatments in the field of child and adolescent psychiatry like MST or MDFT show low to medium effect sizes of *d* = 0.55 (MST, Curtis et al., 2004) or *d* = 0.27–0.77 (MDFT, Liddle et al., 2009). The pre/post comparisons in our study show moderate to high effect sizes with a mean of *d* = 1.04 and a range of *d* = 0.34 – 2.18, which are considerably larger than those reported for MST and MDFT. Even without the ITG, which had considerably larger effect sizes than the other variables, the mean effect size of *d* = 0.7was still at the upper end of the range reported by Liddle et al. (2009).

### Sustained Effects of Treatment

Compared to these therapeutic interventions, the treatment in our study consists of fewer sessions per week but a longer treatment duration. Compared to the mean of 40 sessions in MST and MDFT, the families in our study received a mean of 115 sessions (SD = 19.2) over an average period of 28.8 months. This could hint at treatment effects due to longer treatment duration and dosage. Indeed, Lindfors et al. (2015) showed that long-term therapy resulted in higher and more persistent outcome scores with more ego-structural changes than short-term therapy. Knekt et al. (2017) also showed that long-term therapy was more effective for patients with low-level personality functioning. The duration of therapy matters where structural effects are concerned: Bozzatello and Bellino (2016) showed that structural effects in outpatient therapy for personality disorders persisted in the follow-up period after the termination of therapy. This long-term effect can also be observed in all our variables, in case of GAF, FAI, and two subscales of the CBCL even with further significant improvements.

### Limitations

The study is not a randomized-controlled trial but follows a naturalistic design. In a strict sense, this consequently prevents us from making causal interpretations about the effectiveness of the treatment. However, the main aim here was to evaluate if there was a prolonged effect of the treatment, i.e., the effects would not disappear once the treatment had ended. While “placebo” or “no treatment” control groups do not seem ethically justifiable in this context, future studies should include at least a “waiting list” control group to confirm the assumption of “no change” without treatment that is implied in our analyses.

Although we tried to apply an intention-to-treat analysis instead of a completer analysis, this was not possible because eight families terminated treatment unilaterally and were no longer available for assessment. Still, we included all families who had received treatment for at least 24 weeks.

In addition, there could be a confirmation bias in the data, although the hypotheses of the study were not known to the therapists. We tried to further reduce this by assigning data collection of most variables to external psychologists.

## Conclusion

Our study shows that adaptive disorder-related treatments can achieve sustainable changes in MPFs, which are referred to as “hard to reach.” In addition, the data show that even adolescent non-responders can successfully be treated irrespective of pretreatment characteristics such as low socio-economic status, low level of personality functioning (CBCL dysregulation syndrome), and earlier unilateral termination of treatment.

The present study is a contribution to “treatment aptitude” or “suitability” research (Norcross, 2011) and seeks to improve the adaptive indication (individualization of treatment in psychotherapy) of “tailor made treatments” for MPFs. Klein et al. (2003) showed that it is difficult to change combined disorders of social behavior and dissocial conspicuous behavior in the inpatient setting of youth welfare services. Efficacy studies also point to the low effect sizes in the psychotherapeutic treatment of children and adolescents with combined multimorbid externalizing or dissocial symptoms (Cincaya et al., 2011). Integrated multi-modal treatments with high structural and process qualia can close this supply gap, especially for patients, adolescents, and families with low-level functioning, and achieve medium to high effect sizes and sustainable high follow-up effects.

## Data Availability Statement

The datasets generated for this study are available on request to the corresponding author.

## Ethics Statement

This study was carried out in accordance with the recommendations of the WMA Declaration of Helsinki – Ethical Principles for Medical Research Involving Human Subjects. All subjects gave their written informed consent in accordance with the Declaration of Helsinki. The study received ethics approval from the Government of Salzburg (Amt der Salzburger Landesregierung, Ref. 3/02, Fanny-v.-Lehnert-Strasse 1, 5020 Salzburg, Austria). Further ethics approval from an institutional IRB/Ethics Committee was not required as per applicable institutional and national guidelines and regulations.

## Author Contributions

BA was responsible for statistics, EB for taking care of the clinical psychologists from the data collection team, MN, KV, HS, and HB for the data evaluation, and EB and GS for writing the manuscript. All authors contributed to the study design, literature and references.

## Conflict of Interest

The authors declare that the research was conducted in the absence of any commercial or financial relationships that could be construed as a potential conflict of interest.
