# Supplementary material for: Long-Term Effects of Home-Based Family Therapy for Non-responding Adolescents With Psychiatric Disorders. A 3-Year Follow-Up
Source: Front Psychol. 2020 Oct 23;11:475525. doi: 10.3389/fpsyg.2020.475525 (PMC7644973; doi:10.3389/fpsyg.2020.475525)
Supplement: Supplementary file 1 [file Data_Sheet_1.PDF]

Supplement

Multi-problem Family Collaboration Scale (CP)

The MPF Collaboration Scale is an integral part of routine assessments (Bachler et al., 2013) in the treatment and is assessed by a narrative interview which the therapist conducts with the family. The therapist then estimate the degree of collaboration with the family on a five-point Likert scale: 1) "The family has a deep insight into its problems and shows continuously good goal-directed collaboration." 2) "The family acknowledges itself as being part of the problems and is interested in understanding the problems, and mostly shows willingness to collaborate in a goal-directed manner." 3) "The family shows a passive recognition of its own problems and low to medium goal-directed collaboration." 4) "Problems are experienced as inflicted from the outside; involuntary goal-directed collaboration; working together but feeling forced to." 5) "No insight, complete defense, neglect of problems, goals and goal directed collaboration; no willingness to collaborate." This approach has been shown to be more predictive for outcome rates and to have more homogeneous effect size than an alliance measure which was self-reported by the patients (Bachelor, 2013).

The inter-rater reliability of the MPF Collaboration Scale has previously been shown as ranging from .75-.87 and as correlating at 0.86 with the Heidelberg Structural Change Scale, thus constituting good criterion validity (Bachler et al., 2013).

Treatment Outcome Expectation (VH)

The assessment of the Treatment Outcome Expectation is also part of the routine assessment and is rated by the therapist together with the caregiver and the adolescent on a five-point Likert scale, with high scores indicating low expectations (Bachler et al., 2013).

Child Behavior Checklist (CBCL)

The CBCL (Achenbach, 1991) is a questionnaire for parents to rate the behavior, skills, and problems of their children and adolescents aged 4 to 18 years. The scale is divided into three domains of competence (activity, social competence, school) and eight subscales (somatic, social problems, social withdrawal, anxiety/depression, alertness, schizoid/obsession, dissocial behavior, aggressive behavior). The subscales provide information about externalizing and internalizing behavior, in addition to a total score for problematic behavior. Due to its standardization, the CBCL is objective in terms of implementation, evaluation, and interpretation. Reliability was confirmed in a German clinical sample (n = 1,653) as well as in a field sample (n = 1,622). Good to very good internal consistencies of >.85 were found for overall conspicuousness and the scales "internalizing behavior" and "external behavior". Cronbach's alpha for the total score is .95, and the factorial validity of the subscales was shown by the Working Group on Child, Adolescent and Family Diagnostics (1998). The CBCL is rated by the external psychologist together with the caregiver.

Individual Therapeutic Goals (ITG)

The Individual Therapeutic Goals (ITG) rating follows the ITG module of the Psychotherapy Basic Documentation (PSYBADO, Heuft & Senf, 1998). It provides an individual definition of 3 therapeutic goals that are important both to the family and the Child Welfare Office. The PSYBADO includes five main categories: intrapsychic, interactional, somatic, addictive, and social medicine. The attainment to the therapeutic goals is recorded graphically by the Goal Attainment Scale by the therapist, the family, the child/adolescent, and the Welfare Office. The final score is the average of these ratings. For the Goal Attainment Scale, an inter-rater reliability of .82 has been reported (Steenbeek et al., 2010). Face, construct, and social validity coefficients ranged from .62 to .83 (Winter, Wiegard, Welke, & Lehmkuhl, 2005); the reliability (Cronbach's α) of the PSYBADO is between .65 and .83. The construct validity is reported at .82 (Heuft & Senf, 1998).

Family Adversity Index (FAI)

The Family Adversity Index (Rutter & Quinton, 1977) measures familial psychosocial stress. Based on five items (chronic disharmony in the family, low socioeconomic status, cramped living quarters, parental criminality, and mental disorder of the primary caregiver), the resulting total value ranges from zero to a maximum of five. Values ≥2 reflect considerable socio-familial stress. Reliability was found to be .65 and validity ranged from .66 to .70 (Rutter & Quinton, 1977). The FAI is rated by the by the external psychologist based on the anamnestic information they received.

Mannheim Parental Interview (MPI)

The Mannheim Parental Interview (Esser, Blanz, Geisel, & Laucht, 1989) is a structured and standardized clinical interview, which indicates psychological disorders and their severity. The 37 questions covering child and adolescent psychiatric symptoms combine a cumulative child-psychiatric symptom score and different ICD diagnoses. The interview with the parents was done by the external psychologists. Cronbach's alpha is α = .95, and the percentage of concurrence between professional judgements was 79% (Esser, Blanz, Geisel, & Laucht, 1989).

Global Assessment of Relational Functioning (GARF)

The Global Assessment of Relational Functioning (GARF) rating scale assesses the psychosocial level of family functioning through a clinical interview. It covers the three dimensions problem solving, organization, and emotional climate (Stasch & Cierpka, 2006). The inter-rater reliability is .72 and Cronbach's alpha is .91. The validity coefficients range between .50 and .73 (Denton, Nakonezny, & Burwell, 2010). The GARF is rated by the external psychologist.

Global Assessment of Functioning for Adults and Children (GAF, CGAF)

The Global Assessment of Functioning scale, based on the DSM-IV, is frequently employed in psychotherapy studies as a measure of disability and psychosocial dysfunction (Saß, Wittchen, & Zaudig, 2003). A value of ≤ 50.0 is considered a severe impairment (Parabiaghi, Bonetto, Ruggeri, Lasalvia, & Leese, 2006). Inter-rater reliability scores of .74 have been reported, and Cronbach's alpha

is .92 (Hilsenroth et al., 2000). The questionnaire comes in an adult version (GAF) and a version for children (aged 4 and above) and adolescents (CGAF). The GAF and CGAF is rated by the external psychologist.

Social Self-Sustainability Skills (SSF)

The Social Self-Sustainability Skills Scale records the social self-preservation ability. It is based on the Psycho-DOK system (Bachler, 2013) and consists of five levels: 1 = very good, 2 = good, 3 = slightly restricted, 4 = clearly restricted, and 5 = massively restricted. The construct describes – independently of social support systems – factors such as social assistance, working capacity in the family, and the family income earned through this work. The SSF is rated based on a checklist by the external psychologist.

Social and Working Skills (SSAMJ)

The SSAMJ records the school and work ability of the minors and defines the extent to which age-appropriate social behavior and performance can be achieved (Bachler, 2013). It contains 5 levels: 1 = good, 2 = slightly impaired, 3 = clearly impaired, 4 = severely impaired, 5 = impossible. The SSAMJ is rated by the external psychologist.

REFERENCES

Achenbach, T. M. (1991). *Manual of the Child Behavior Checklist 4/18 and 1991 Profile.* Burlington: University of Vermont, Department of Psychiatry.

Bachelor, A. (2013). Clients' and therapists' views of the therapeutic alliance: similarities, differences and relationship to therapy outcome. *Clin Psychol Psychother.; 20*(2), 118-135. doi: 10.1002/cpp.792

Bachler, E. (2013). *Familien-therapeutische Interventionen (TAF) bei Multiproblemfamilien. Effektstärken (ES) und klinische Signifikanz (CS) von therapeutischem „Home-based treatment" (long term). Eine Prozess-Ergebnis-Studie. [Family therapy interventions (TAF) for multi-problem families. Effect sizes (ES) and clinical significance (CS) of therapeutic "home-based treatment" (long term). A process-outcome study].* Dissertation Paracelsus Medizinischen Privatuniversität, Salzburg.

Denton, W. H., Nakonezny, P. A. & Burwell, S. R. (2010). Reliability and validity of the global assessment of relational functioning (GARF) in a psychiatric family therapy clinic. *J Marital Fam Ther.; 36*(3), 376-387.

Esser, G., Blanz, B., Geisel, B. & Laucht, M. (1989). *Das Mannheimer Elterninterview.* Weinheim: Beltz.

Heuft, G., and Senf, W. (1998). *Praxis der Qualitätssicherung in der Psychotherapie: Das Manual zur PSYBADO. Entsprechend den Empfehlungen der psychotherapeutischen Fachgesellschaften. [Practice of quality assurance in psychotherapy: The Manual for PSYBADO. According to the recommendations of psychotherapeutic societies].* Stuttgart: Thieme.

Hilsenroth, M. J., Ackerman, S. J., Blagys, M. D., Baumann, B. D., Baity, M. R., Smith, S. R., et al. (2000). Reliability and validity of DSM-IV axis V. *Am J Psychiatry.;157*(11), 1858-1863.

Parabiaghi, A., Bonetto, C., Ruggeri, M., Lasalvia, A. & Leese, M. (2006). Severe and persistent mental illness: a useful definition for prioritizing community-based mental health service interventions. *Soc Psychiatry Psychiatr Epidemiol, 41*(6), 457-463.

Rutter, M. & Quinton, D. (1977). Psychiatric disorder ecological factors and concepts of causation. In M. McGurk (Ed.). *Ecological factors in human development*. (pp. 173-187). Amsterdam: North Holland.

Saß, H., Wittchen, H. U., Zaudig, M., & Houben, I. (2003). *Diagnostische Kriterien des diagnostischen und statistischen Manuals psychischer Störungen.* DSMIV-TR. Hogrefe, Göttingen.

Stasch, M. & Cierpka, M. (2006). Beziehungsdiagnostik mit der GARF-Skala. *Psycho-therapeut,11*, 56-63.

Steenbeek, D., Ketelaar, M., Lindeman, E., Galama, K. and Gorter, J.W. (2010). Interrate reliability of goal attainment scaling in rehabilitation of children with cerebral palsy. *Archives of Physical Medical Rehabilitation, 91*(3), 429-435.

Winter, S., Wiegard, A., Welke, M. & Lehmkuhl, U. (2005). [Evaluation with the "Psychotherapie Basisdokumentation" for Children and Adolescents: Psy-BaDo-KJ – a questionnaire for quality assurance and evaluation of psychotherapy for children and adolescents]. *Z Kinder Jugendpsychiatr Psychother., 33*(2), 113-122.
